# Caring for patients during voluntarily stopping of eating and drinking (VSED): experiences of a palliative care team in Germany

**DOI:** 10.1186/s12904-023-01308-z

**Published:** 2023-11-21

**Authors:** Yann-Nicolas Batzler, Manuela Schallenburger, Pia Maletzki, Theresa Tenge, Daniel Schlieper, Jacqueline Schwartz, Martin Neukirchen

**Affiliations:** 1 Interdisciplinary Centre for Palliative Medicine, Medical Faculty and University Hospital Düsseldorf, Heinrich Heine University Düsseldorf, Düsseldorf, Germany; 2 Department of Anaesthesiology, Medical Faculty and University Hospital Düsseldorf, Heinrich Heine University Düsseldorf, Düsseldorf, Germany; 3 Interdiscipilinary Centre for Palliative Medicine, Medical Faculty and University Hospital Düsseldorf, Heinrich Heine University Düsseldorf, Centre for Integrated Oncology Aachen Bonn Cologne Düsseldorf (CIO ABCD), Düsseldorf, Germany

**Keywords:** Palliative care, Voluntarily stopping of eating and drinking (VSED), Moral distress, Resilience, Multi-professional team

## Abstract

**Background:**

Health-care professionals are confronted with patients who wish to end their lives through voluntarily stopping eating and drinking (VSED). During VSED, symptoms such as agitation, thirst or psychological distress may arise, thus making close medical accompaniment necessary. Dealing with these symptoms can put a high burden on palliative care teams. Furthermore, divergent perceptions of the ethical classification of VSED may lead to moral distress. The aim of this study was to assess the influence of experience gained over time on the burden of palliative care professionals while accompanying patients during VSED and to assess the perceptions of coping strategies.

**Methods:**

This is a prospective single-centre study conducted at the Interdisciplinary Centre for Palliative Care at University Hospital Duesseldorf, Germany. At two points in time (T1, T2) one year apart, team members of all professions who were actively involved in the accompaniment were eligible to complete a pretested questionnaire.

**Results:**

Team members perceived the symptom complex of psychological distress, anxiety, and agitation to be the most burdensome symptoms for the patients (T1: 28/49, 57.1%; T2: 33/59, 55.9%). Thirst was the second most observed symptom (T1: 17/49, 34.7%, T2: 19/59, 32.2%). These were also the most burdensome symptoms for individual team members. Most team members found there were no general moral concerns. There was a decrease in the perceived importance of support strategies such as ethical counselling (85.7% versus 63.6%).

**Conclusions:**

Accompanying patients during VSED is a challenge for health-care professionals. When comparing T2 to T1, less emphasis lies on the importance of ethical counselling or psychiatric assessment to build a foundation for the accompaniment. Moral and ethical concerns seem to play a minor role. More in-depth studies covering a bigger sample size as well as qualitative studies are needed.

**Supplementary Information:**

The online version contains supplementary material available at 10.1186/s12904-023-01308-z.

## Background

In palliative care as well as in all medical disciplines, health-care professionals (HCP) are repeatedly confronted with patients who wish to hasten death [1, 2]. These wishes may be multi-faceted and may arise due to a patient’s high symptom burden, loss of perspective in life, not wanting to be a burden on one’s family or out of fear of an agonizing dying process [2–4]. Being able to openly speak about these wishes is often a challenge for patients, as is dealing with them appropriately on the side of HCP [2, 5]. In Germany, discussions about different ways of hastening death have increased after paragraph 217 of the German criminal code was abolished in 2020 [6]. This law prohibited rendering suicide assistance on a recurring basis [7]. However, there are insecurities on the side of both patients and HCP in regard to dealing with this in daily clinical practice. Up to this date, definite regulations of assistance with suicide are non-existent in Germany [8]. Voluntarily stopping of eating and drinking (VSED) can be an alternative to assisted suicide [3, 6]. VSED implies that competent patients stop eating and drinking to hasten their death [1, 9–11]. In Switzerland, approximately 0.5–0.7% of all deaths are attributable to VSED [11]. The process of VSED until death may take up to 21 days [12]. VSED is described as a sometimes burdensome process due to thirst, pain or delirium that may arise [10, 13]. Furthermore, next of kin may be confronted with a lot of distress by both accompanying their loved ones and accepting the end-of-life decision [11, 14]. All these factors contribute to a high burden on HCP teams when accompanying patients during VSED [11]. Next to somatic and social dimensions of suffering, psychological and spiritual needs need special focus. Furthermore, patients, next of kin and medical teams are confronted with existential crises Which require them to engage in thorough and repeated reflection in order to cope sufficiently [11]. This situation is further aggravated as perspectives on the ethical and moral dimensions surrounding VSED may differ substantially within HCP teams [6, 15, 16].

In the literature, VSED is regarded as a form of suicide, a physiological way of dying or an action of its own kind (*sui generis*) [8, 11, 17–19]. In Switzerland, as an example, most HCP regard VSED as a natural way of dying [13, 20]. This classification, however, is important as it leads to classifications concerning the accompaniment of patients during VSED: It might be regarded as a form of assisted suicide by some HCP [18, 21]. On the other hand, accompaniment might be seen as an obligation towards the patient in order to alleviate an otherwise burdensome process [8, 15, 17]. The individual attitude towards VSED is a result of one’s own personal and professional experiences, one’s knowledge of the law, patients’ age, type of disease and the patients differentiated and transparent decision to engage in VSED [22].

As a result, divergent views may lead to conflicts within the teams, but also to moral distress for individual team members [22, 23]. Moral distress is defined as: “One knows the ethically correct thing to do, but is prevented from acting on that perceived obligation” [24].

To support HCP, the German Association for Palliative Care (Deutsche Gesellschaft für Palliativmedizin, DGP) published a statement on VSED [25]. It is stated that an in-depth assessment before deciding on accompanying patients during VSED is crucial [9, 25]. The somatic, social, spiritual and psychological dimensions of suffering need to be addressed, and options of symptom control should be offered repeatedly [10]. Patients should be assessed in regard to legal capacities and power of judgement [26]. As mechanisms to build resilience within the team, the opinion of each member should be respected and repeated internal discussions [11] as well as case and ethical discussions should take place [25]. Whenever a patient at our palliative care centre seeks aide in VSED, timely ethical counselling, psychiatric assessment, repeated team meetings and case supervision take place.

In order to gain a deeper insight into the implications of the process of VSED, this study was conducted in order to assess burdensome symptoms, ethical and moral perceptions of HCP as well as to evaluate different coping strategies. Furthermore, it was intended to assess whether an increase in experience over time influences these factors.

## Methods

### Study design and ethical approval

This study is a prospective single-centre study conducted at University Hospital Duesseldorf, Germany. The aim was to assess both the burdens on medical staff whilst accompanying patients during VSED at two points in time (T1: Q4 2021, T2: Q4 2022) and whether a gain of experience over time leads to different perceptions of VSED and coping strategies. After the ban on assisted suicide services was lifted by the German Federal Constitutional Court in 2020, we saw a rising interest in medical aid in dying T1 was defined as the point in time at which we accompanied a first patient during VSED who was admitted to our ward for this purpose after repeated counselling via telephone. T2 was defined as T1 + 12 months. Between T1 and T2, our team accompanied two more patients during VSED.

Ethical approval was obtained by the local ethics committee (reference number 2021 − 1490_1).

### Data collection and measures

For the purpose of this study, a questionnaire was designed by the study team and developed in interdisciplinary and multi-professional discussions in the Interdisciplinary Centre for Palliative Care Medicine. The questionnaire was designed in accordance to a validated questionnaire from a Swiss study: questions were adapted (e.g. ethical classification of VSED, quality of death), others added for this study’s purpose (e.g. perception of the relevance of ethical counselling, team supervision or change of attitude towards VSED after the accompaniment), and, for T2, more questions from the validated questionnaire were added (e.g. relevance to work and institution’s culture) [20]. The questionnaire was handed out in German and can be found in the Supplementary Materials (translated back into English, “Questionnaire”). The questionnaire was pretested in repeated rounds as well as pilot tested at the Interdisciplinary Centre for Palliative Care at University Hospital Duesseldorf and adapted according to the feedback obtained before the study start (e.g. multiple answer options to name burdensome symptoms, adaption of age strata to ensure anonymity).

Both questionnaires use Likert-scale like responses to reflect on moral perception of VSED (1 = strongly disagree, 2 = disagree, 3 = neutral, 4 = agree, 5 = strongly agree). The study site’s questionnaire included an item to reflect on the burdensome symptoms of patients (this was the only item that allowed for multiple answers), one open question to assess the most burdensome symptom of team members (single answer), as well as dichotomous answer options to reflect on coping strategies that were offered to the team. Demographic data was assessed using categorical variables.

All team members (n = 52) that were actively involved in the treatment of symptoms during VSED were asked to participate in this study. During the accompaniment of the patient at T1, all professions that work at the Interdisciplinary Centre for Palliative Care at University Hospital Duesseldorf were contacted via email and informed about the study. Furthermore, they were invited to take part in the study during daily team meetings. Team members were able to participate in the study for up to two weeks after the patient passed away. For T2, team members could take part in the study during a time period of two weeks twelve months after T1. After one week, a reminder email to participate was sent.

Eligible professions were physicians, nurses, physiotherapists, spiritual counsellors, clerks, social workers, hospice volunteers, and psychologists. The only inclusion criterion was taking part in the accompaniment (e.g., daily ward rounds, symptom management, physiotherapy, massages, spiritual or psychological counselling). Team members who did not play an active role were excluded. In total (T1 and T2), there were 53 members of the team eligible to participate.

### Statistical analysis

Data analysis was performed using Microsoft Excel 2020 (version 16.42, Microsoft Corp., Redmond, WA, USA), STATA (version 17.0, StataCorp, College Station, TX, USA) and IBM SPSS Statistic (version 28.0.1.1, IBM, Armonk, NY, USA). Professions as well as symptoms were tested for normality using Shapiro-Wilk test due to the small sample size. To compare answers at T1 and T2, a non-parametric Mann-Whitney U-test was used based on the results of the Shapiro-Wilk test. Contingency tables and Exact Fisher’s test were used to test for significance while p < 0.05 was considered as statistically significant. Continuous variables are presented by the mean and standard deviation (SD), categorical variables are shown as absolute and relative (percentage) frequencies. Due to the explorative character of this study p-values were not adjusted.

## Results

### Demographics

Between T1 and T2, four patients were accompanied during VSED. Demographic data is summarized in Table 1. At each measurement time, 22 team members took part in the study (response rate 41.5%). At T2, 12 team members already participated in the first measurement (54.5%). There were no statistically significant differences when analysing the results of the 12 participants who took part at both points in time.

At both points in time, nurses were represented most, followed by physicians. Few members of other professions participated.

### Symptom burden

The participants assessed the symptoms causing the greatest burden on patients (multiple answers possible). In total, the 22 participants gave 49 answers at T1 and 39 at T2.

At T1, 18/22 participants (81.8%) anticipated the observed symptoms to occur before VSED was initiated whereas at T2, 90.9% (20/22) participants anticipated them. Team members perceived the symptom complex of psychological distress, anxiety, and agitation to be the most burdensome symptoms for patients (T1: 28/49, 57.1%; T2: 33/59, 55.9%). Thirst was the second most observed symptom by team members (T1: 17/49, 34.7%, T2: 19/59, 32.2%). Symptoms that were only rarely observed were hunger, dyspnea, and pain (T1: 4/49, 8.2%, T2: 7/59, 11.9%).

During the accompaniment, team members experienced stress while trying to treat symptoms adequately. Throughout all professions, psychological distress, anxiety and agitation were perceived as most difficult to treat at T1 (10/22, 45.4%), followed by thirst (5/22, 22.7%). Seven team members (31.8%) did not experience any symptoms as burdensome on themselves. At T2, the most burdensome symptom that team members had to deal with was thirst (14/22, 63.3%) followed by psychological distress and agitation (8/22, 63.7%).

A subgroup analysis was performed to compare nurses and physicians. At T1, 5/9 (55.5%) nurses felt psychological distress, agitation, and anxiety to be the most challenging symptoms to deal with, whereas physicians (4/7, 57.1%) mostly perceived no observed symptom as burdensome (Exact Fisher’s Test: *p* = 0.5). At T2, 10/13 nurses (76.9%) felt that thirst was a challenging symptom to deal with, physicians felt both thirst (3/6, 50.0%) and psychological distress (3/6, 50.0%) affecting themselves (Exact Fisher’s test: *p* = 0.3). Professions and their experience of burdensome symptoms were statistically not significantly related.

Overall, at T2, participants felt that the team was burdened while accompanying patients during VSED (11/6, 50.0%; neutral: 6/22, 27.3%; disagree: 5/22, 22.7%), even though this was not further distinguished. In general, participants felt that the patients were granted a death with dignity (T1: 22/22, 100.0%; T2: 21/22, 95.5%).

**Table 1:** Demographics

			T1								T2			
Factor							Answers, n (%)							
Profession	*Nurses*	*Physiscians*	*PS*	*PT*	*Volunt.*	*SC*	*Clerk*	*Nurses*	*Physiscians*	*PS*	*PT*	*Volunt.*	*SC*	*Clerk*
	9 (40.9)	7 (31.8)	2 (9.1)	1 (4.5)	1 (4.5)	0 (0.0)	2 (9.1)	13 (59.1)	6 (27.3)	1 (4.5)	1 (4.5)	0 (0.0)	1 (4.5)	0 (0.0)
Sex	*Female*	*Male*	*Diverse*					*Female*	*Male*	*Diverse*				
	14 (63.6)	8 (36.4)	0 (0.0)					17 (77.3)	5 (22.7)	0 (0.0)				
Age	*< 20*	*20–30*	*31–40*	*41–50*	*51–60*	*61–70*		*< 20*	*20–30*	*31–40*	*41–50*	*51–60*	*61–70*	
	0 (0.0)	4 (18.2)	6 (27.3)	5 (22.7)	6 (27.3)	1 (4.5)		0 (0.0)	1 (4.5)	7 (31.8)	8 (36.4)	4 (18.2)	2 (9.1)	
Work in PC	*< 1 year.*	*1–5 year.*	*6–10 year.*	*> 10 year.*				*< 1 year*	*1–5 year.*	*6–10 year.*	*> 10 year.*			
	6 (27.3)	7 (31.8)	4 (18.2)	5 (22.7)				4 (18.2)	9 (40.9)	4 (18.2)	5 (22.7)			
Experience in VSED beforehand	*Yes*	*No*						*Yes*	*No*					
	16 (72.7)	6 (27.3)						22 (100.0)	0 (0.0)					
Patients accompanied beforehand	*0*	*< 5*	*≥ 5*					*0*	*< 5*	*≥ 5*				
	6 (27.3)	13 (59.1)	3 (13.6)					0 (0.0)	15 (68.2)	7 (31.8)				

yr.: years; PS: Psychology, PT: Physical Therapy/Massage, Volunt.: Volunteers, SC: Spiritual Care

### Ethical and moral perceptions

**Fig. 1:**
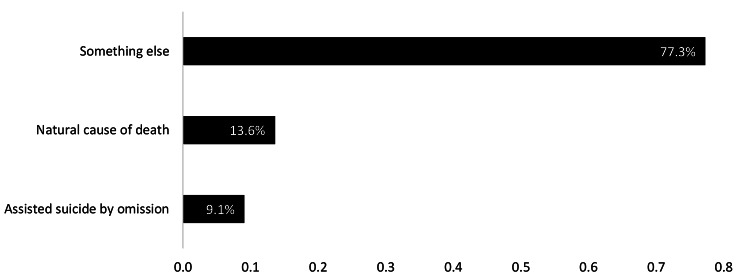
Ethical classification of voluntarily stopping of eating and drinking (VSED). Most team members perceive VSED to be “something else”/ a category of its own kind

In general, participants indicated (at T2) that VSED was very relevant to their work (21/22, 95.5%). Most participants stated that VSED was not contradictory to the institution’s culture (18/22, 81.8%) and that VSED was compatible with their world view or religion (18/22, 81.8%). Figure 1 shows the ethical classification of VSED at T2. Subgroup analysis for physicians and nurses showed no statistically significant relation between profession and classification (p = 0.2).

**Fig. 2:**
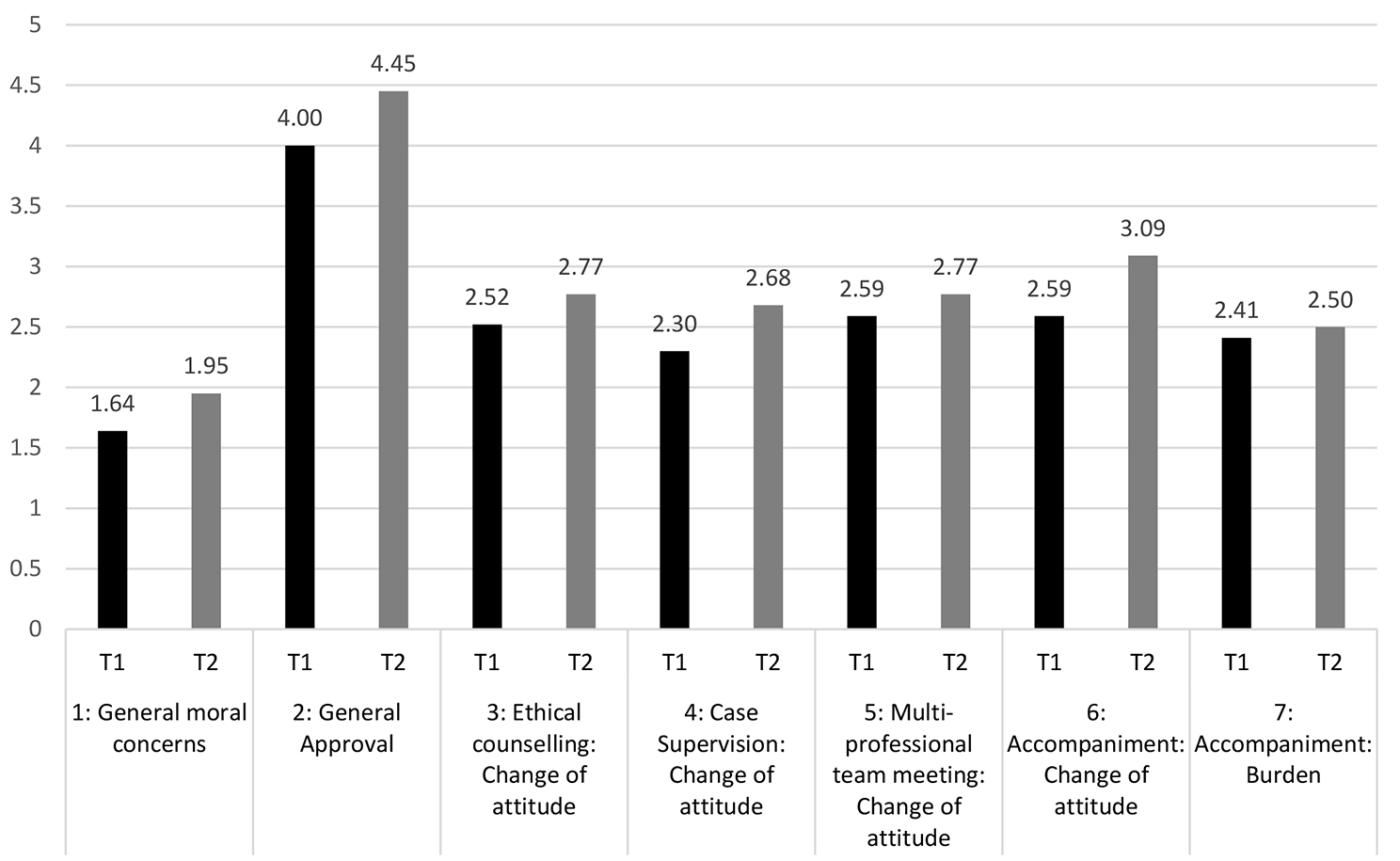
Moral perception and change of attitude (mean values). 1: I have moral doubts regarding VSED; 2: I would generally accept to accompany patients during VSED; 3: The ethical counselling changed my attitude towards VSED; 4: The case supervision changed my attitude towards VSED; 5: The multi-professional team meeting changed my attitude towards VSED; 6: The accompaniment changed my attitude towards VSED; 7: During VSED, professionals are morally burdened

Figure 2 shows the results regarding the moral perception and changes of attitude in team members throughout ethical counselling, case supervisions, team meetings and accompaniments in general. Overall, there were only small differences between T1 to T2. At T2, the accompaniment in general gained the highest mean value in regard to change of attitude (T1: SD 1.10; T2: SD 1.31). Overall, the accompaniment of patients was not perceived as morally burdensome (T1: SD 1.01; T2: SD 1.26). Mann-Whitney U test showed no statistical significance in the perceptions between T1 and T2. The results are shown in Table 2.

**Table 2:** Mann-Whitney U test for moral perception and change of attitude over time

Item	z-Score	p Value
General moral concerns	-0.94	0.35
General approval	-1.04	0.30
Ethical counselling: Change of attitude	0.55	0.58
Case supervision: Change of attitude	1.10	0.27
Multi-professional team meeting: Change of attitude	-0.53	0.60
Accompaniment: Change of attitude	-1.15	0.25
Accompaniment: Moral Burden	-0.04	0.97

### Coping strategies

**Fig. 3:**
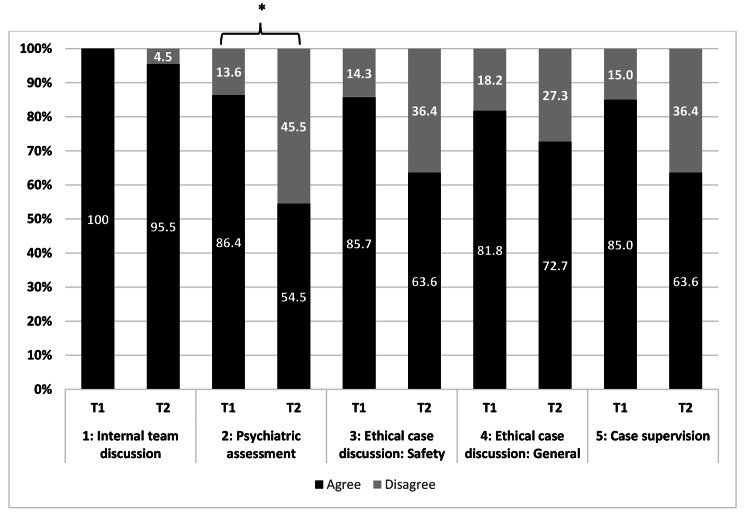
Evaluation of coping strategies (%, * = p < 0.05). 1: The internal team discussion offered enough space and safety to discuss uncertainties and worries in regards to a possible accompaniment of VSED patients. 2: The specialist psychiatric assessment of the patients’ ability to consent helped me in accepting the patients’ wish to hasten death. 3: After the ethical case discussions, I felt safer in accompanying the patients during VSED. 4: In general, ethical case discussions should take place before deciding on accompanying patients during VSED. 5: The case supervision helped me in coping with the experiences made during the process of VSED

Figure 3 shows the results of the study participants’ assessment of coping strategies for both T1 and T2. In general, at T1, agreement was higher than at T2, especially regarding the importance of psychiatric assessment beforehand (86.4% versus 54.5%). In contrast, 17/22 (77.3%) participants stated that it is important to determine the patients’ ability to judge the situation at T2. Furthermore, ethical case discussions were regarded more important at T1 than they were at T2 (85.7% versus 63.6%). Only item 2 showed a statistically significant relation (*p*=0.045).

## Discussion

This is one of the first studies in Germany to assess HCP’s perceptions of VSED, its related burdens and possible coping strategies.

The discussions regarding assisted suicide are still ongoing in Germany, even more after the ban on assisted suicide services was lifted in 2020. So far, there are no concrete regulations on how to deal with a patient’s wish to hasten their death. In particular, VSED is a distinct form of ending one’s own life as there is also an ongoing discussion on how to perceive VSED. Possible perceptions are: a form of suicide, a natural way of dying or an action of its own [9, 15, 26]. For HCP, this ultimately leads to the question whether accompanying patients during VSED is a form of assisted suicide, e.g., in the form of assisted suicide by omission [8]. This moral dilemma is then aggravated through severe symptoms that may arise during VSED, leading to even more distress for HCP [11].

In our study, the symptoms which were perceived to be most burdensome differed between both points in time: At T1, the symptom complex of psychological distress, anxiety and agitation was the most burdensome, at T2, it was thirst. This implies that occurring symptoms may not be generalised but should be assessed for each individual patient. Team members stated that treating severe symptoms during VSED is a burdensome process, which was not linked to specific professions. There was also no correlation to experiences made beforehand, e.g. in the form of a decline of stressful symptom management. Every single team member may experience difficulties and challenging moments during the accompaniment. While there are also HCP who do not experience any burden, those who feel a burden must be given enough space to reflect on the process. In our cohort, as well as in the literature, all participants felt that the patients were granted a death with dignity [13] which may be a factor which alleviates distress. Furthermore, this implies the importance of professional accompaniment during VSED through specialized HCP to ensure a symptom-controlled passing.

As the perceived symptoms were regarded burdensome, moral concerns rarely occurred. HCP in this study stated that they generally approve VSED. This was supported by the fact that there was no change of attitude regarding the moral perception of VSED after discussing this topic in ethical counselling, case supervisions and internal team meetings. During the study period, there was no significant relation between experiences and change of attitudes, and personal opinions differed only slightly. Furthermore, VSED did not seem to oppose to participants’ world view or religion. An underlying reason might be that HCP mainly perceived VSED to be an action of its own and not as suicide or a natural dying process in contrast to the literature [11]. This classification of VSED is in accordance with the perception of the German Association for Palliative Care [24]. Only few team members felt that the accompaniment was a form of assisted suicide by omission. This, however, leaves room for individual perceptions and highlights the importance of ongoing research in this topic in order to further assess specific implications surrounding VSED.

The internal team discussion was perceived as helpful in addressing problems as it offered a safe space for HCP. This finding is in accordance with previous work [13]. The importance of psychiatric counselling to assess the patients’ ability to consent experienced the highest decrease in importance over time which was also statistically significant when comparing T1 and T2. A possible explanation for this might be the experience of HCP in judging patients’ ability to consent over time through various informed consent processes. This seems to not make psychiatric assessment a needed tool in order to build a moral legitimation. Ethical case discussions build a solid moral ground for team members and should be part of any decisions during VSED. Especially for unexperienced HCP, this may contribute to an ethical foundation during the accompaniment. After the accompaniment, specific case supervisions with experts help to reflect on challenging situations [11]. At T1, they were perceived very important, and, like all items, were not as important to HCP at T2, most probably due to a higher rate of experience.

### Limitations

Our study design has several limitations. First, the study team’s part of the questionnaire was not validated, however, it was highly influenced by a validated Swiss questionnaire (Incidence and Attitudes to Voluntary Stopping of Eating and Drinking) [11, 13, 20]. Second, the small number of study participants led to insignificant results in statistical analysis. Therefore, our results can only serve as an approximation to the delicate topic surrounding VSED. Third, not all team members who took part at T1 also participated at T2 which influences the comparison between the two points in time (e.g., in regards to knowledge gains, shifts in perceptions, reduced burden etc.). Fourth, this study only took place at one study site. It would be interesting to assess if perceptions differ among organizational standards or regulations. In addition, there might be confounding factors such as religion, spirituality, age and experience that could have led to bias which, due to the small study population, were not taken into consideration in the statistical analysis.

### Outlook

This study serves as a first approach to determine the burden which is imposed on HCP during the accompaniment of patients during VSED in Germany. The results could contribute to the current political and population-centred discussions on different options of self-determined death. We were able to work out core elements that seem to be important when dealing with VSED, however, this is a very delicate topic entailing personal nuances. We therefore decided to further approach this topic in a qualitative study in the sense of a mixed methods design: A Framework analysis with semi-structured interviews is being developed with the focus on physicians and nurses to dwell deeper into the actual reasons for the depicted burdens within this study.

### Electronic supplementary material

Below is the link to the electronic supplementary material.

Supplementary Material 1

## Data Availability

All data generated or analysed during this study are included in this published article and its supplementary information files.

## Abbreviations

HCP: Healthcare professional
PC: Palliative Care
PS: Psychology
PT: Physical Therapy/Massage
SC: Spiritual Care
SD: Standard deviation
Volunt.: Volunteers
VSED: Voluntarily stopping of eating and drinking
