# Supplementary material for: Caring for patients during voluntarily stopping of eating and drinking (VSED): experiences of a palliative care team in Germany
Source: BMC Palliat Care. 2023 Nov 21;22:185. doi: 10.1186/s12904-023-01308-z (PMC10662259; doi:10.1186/s12904-023-01308-z)
Supplement: Supplementary file 1 — Supplementary Material 1 [file 12904_2023_1308_MOESM1_ESM.docx]

Voluntarily Stopping of Eating and Drinking (VSED)

– Experiences of a multi-professional Palliative Care team –

General Information

**Profession (#):**

○ Nurse

○ Physician

○ Psychologist

○ Physical therapist/ massage

○ Social worker

○ Spiritual Care

○ Volunteer

○ Clerk

**Sex (#):**

○ Female

○ Male

○ Diverse

**Age (#):**

○ 20-30

○ 31-40

○ 41-50

○ 51-60

○ 61-70

**Work experience in Palliative Care (#):**

○ < 1 year

○ 1-5 years

○ 6-10 years

○ > 10 years

**I have accompanied patients during VSED.**

○ Yes

○ No

**Approximately how many cases of VSED have you cared for since the beginning of your working career (#)?**

○ < 5

○ ≥ 5

Symptom management

**The main symptoms during VSED were (multiple answers possible) (#):**

○ Hunger

○ Thirst

○ Dyspnea

○ Agitation

○ Fear

○ Psychological distress

○ Pain

**Which of these symptoms was the most burdensome on you during the accompaniment (single answer)?**

○ Hunger

○ Thirst

○ Dyspnea

○ Agitation

○ Fear

○ Psychological distress

○ Pain

**I expected these symptoms to occur.**

○ Yes

○ No

**The death was dignified (#).**

○ Yes

○ No

Coping strategies

**The multi-professional team meetings offered enough room to talk about uncertainties and worries in regards to the accompaniment.**

○ Yes

○ No

**The psychiatric assessment helped me in accepting the patients’ wish for VSED.**

○ Yes

○ No

**After the ethical counselling, I felt safer in the accompaniment during VSED.**

○ Yes

○ No

**In general, ethical counselling should take place.**

○ Yes

○ No

**The case supervision helped me in coping with the experiences made during the accompaniment.**

○ Yes

○ No

Personal perception

**I have moral doubts regarding VSED (*).**

○ Strongly Agree

○ Agree

○ Neutral

○ Disagree

○ Strongly disagree.

**VSED is compatible with my way of viewing the world or religion (*).**

○ Strongly Agree

○ Agree

○ Neutral

○ Disagree

○ Strongly disagree.

**Which of the following opinions regarding explicit VSED do you agree with (#)?**

○ VSED is to be equated with medically assisted suicide.

○ VSED is to be equated with “leaving to die”.

○ VSED is a natural death along with medical and nursing support.

○ VSED is something else.

**I would generally accept to accompany patients during VSED (#).**

○ Strongly Agree

○ Agree

○ Neutral

○ Disagree

○ Strongly disagree.

**The determination of the sagacity of those affected with VSED is crucial (*).**

○ Strongly Agree

○ Agree

○ Neutral

○ Disagree

○ Strongly disagree.

**The ethical counselling changed my attitude towards VSED.**

○ Strongly Agree

○ Agree

○ Neutral

○ Disagree

○ Strongly disagree.

**The case supervision changed my attitude towards VSED.**

○ Strongly Agree

○ Agree

○ Neutral

○ Disagree

○ Strongly disagree.

**The multi-professional team meeting changed my attitude towards VSED.**

○ Strongly Agree

○ Agree

○ Neutral

○ Disagree

○ Strongly disagree.

**The accompaniment changed my attitude towards VSED.**

○ Strongly Agree

○ Agree

○ Neutral

○ Disagree

○ Strongly disagree.

**During VSED the professionals are morally burdened. (*)**

○ Strongly Agree

○ Agree

○ Neutral

○ Disagree

○ Strongly disagree.

**How would you judge the relevance of VSED for your daily work? (*)**

○ Very relevant

○ Relevant

○ Less relevant

○ Not relevant

○ Do not know

* Taken from: Stängle S, Schnepp W, Mezger M, Büche D, Fringer A. Development of a Questionnaire to Determine Incidence and Attitudes to “Voluntary Stopping of Eating and Drinking.” SAGE Open Nursing. 2019;5

# Adapted from: Stängle S, Schnepp W, Mezger M, Büche D, Fringer A. Development of a Questionnaire to Determine Incidence and Attitudes to “Voluntary Stopping of Eating and Drinking.” SAGE Open Nursing. 2019;5
